# Occurrence and abundance of a *mariner*-like element in freshwater and terrestrial planarians (Platyhelminthes, Tricladida) from southern Brazil

**DOI:** 10.1590/S1415-47572009005000072

**Published:** 2009-12-01

**Authors:** Fernanda Sperb, Desirée Cigaran Schuck, Jaqueline Josi Samá Rodrigues

**Affiliations:** Laboratório de Biologia Molecular, Universidade do Vale do Rio dos Sinos, São Leopoldo, RSBrazil

**Keywords:** *mariner*, planarian, transposable element, transposons

## Abstract

Transposable elements are DNA sequences present in all the large phylogenetic groups, both capable of changing position within the genome and constituting a significant part of eukaryotic genomes. The *mariner* family of transposons is one of the few which occurs in a wide variety of taxonomic groups, including freshwater planarians. Nevertheless, so far only five planarian species have been reported to carry *mariner*-like elements (MLEs), although several different species have been investigated. Regarding the number of copies of MLEs, *Girardia tigrina* is the only planarian species in which this has been evaluated, with an estimation of 8,000 copies of the element per haploid genome. Preliminary results obtained in our laboratory demonstrated that MLE is found in a large number of different species of planarians, including terrestrial. With this in mind, the aim was to evaluate the occurrence and estimate the number of MLE copies in different planarian species collected in south Brazil. Twenty-eight individuals from 15 planarian species were analyzed. By using PCR and the hybridization of nucleic acids, it was found that MLE was present in all the analyzed species, the number of copies being high, probably over 10^3^ per haploid genome.

## Introduction

**Figure 1:**
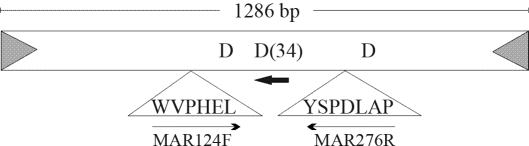
Structure of the *mariner* family element and annealing region of the primers used in this study. Light gray triangles: 28 bp inverted terminal repeats; unshaded box: *mariner* transposase encoding region and corresponding positions of the D, D(34) D conserved motif of the catalytic domain; triangles below the box: position of WVPHEL and YSPDLAP transposase conserved domains and where MAR124F and MAR276R primers were designed; dark-gray arrow: annealing region of reverse primers specific to *cecropia* and *mellifera* subfamilies used in the semi-nested PCR combined with the MAR124F primer.

Transposable elements are DNA sequences capable of changing their position within the genome, and which constitute a significant part of eukaryotic genetic material. The *mariner* family is a diverse and taxonomically widespread group of Class II transposable elements, characterized by possessing inverted terminal repeats flanking their sequences and through codifying a transposase which presents the D,D(34)D motif in its catalytic domain (Figure 1). The transposition of this element takes place by using the conservative “cut-and-paste” mechanism (reviewed in Hartl *et al.*, 1997a). All the transposition steps are executed by this transposase, this being sufficient for element mobility since there is no need for host factors (Lampe *et al.*, 1999; Tosi and Beverley, 2000). *Mariner*-like elements (MLEs) have been classified into 13 different subfamilies, according to nucleotide sequence similarities, and can coexist within one and the same genome (Robertson and MacLeod, 1993; Hartl *et al.*, 1997b; Robertson, 1997; Kumaresan and Mathavan, 2004; Zakharkin *et al.*, 2004). The five major subfamilies are: *capitata*, *cecropia, irritans, mauritiana* and *mellifera* (Lampe *et al.*, 2001). Sequence identity among members of these different subfamilies ranges from 40 to 56% for DNA and 23 to 45% for amino acids (Robertson, 1993).

The assignment of each sequence to a particular subfamily is generally unambiguous. Phylogenetic analysis results in the division of sequences into the same groupings, thereby reinforcing the confidence in assigning these to particular subfamilies and in their reality as being evolutionarily distinct clades (Robertson and MacLeod, 1993).

*Mariner* family elements were first described in *Drosophila mauritiana* as being an insertion of the *white* gene (Jacobson and Hartl, 1985). Since then, they have been identified in plants, fungi, vertebrate animals including man, nematodes, several insect orders and other arthropods (Bigot *et al.*, 1994; Auge-Gouillou *et al.*, 1995; Robertson, 1995; Smit and Riggs, 1996; Robertson, 1997; Plasterk *et al.*, 1999; Feschotte and Wessler, 2002). MLEs have already been identified in five species of the phylum Platyhelminthes, all sequences having been deposited in GenBank at NCBI. Four of these belong to the planarian group (Garcia-Fernàndez *et al.*, 1995; Robertson, 1997; Alvarado *et al.*, 2002). On studying10 species of freshwater planarians, Garcia-Fernàndez *et al.* (1995) were able to identify an MLE only in *Girardia tigrina,* with an estimated 8,000 copies of the element per haploid genome. Nevertheless, they did not encounter this element in the phylogenetically very close species *Schmidtea mediterranea,* whereas more recently, Alvarado *et al.* (2002) did. Robertson (1997) also detected the presence of MLE in *G. tigrina*, as well as in the two species of marine planarians *Bdelloura candida* and *Stylochus zebra.* These results raised the question of the presence of this element in planarians where sporadic distribution among related species can also be observed. The *mariner* family as a whole can be transmitted horizontally between reproductively isolated or phylogenetically unrelated species, as is the case of other transposable elements. This possibility explains the sporadic distribution of this transposon and the great similarity in *mariner* sequences found in species from very different phyla (Robertson, 1993; Robertson and MacLeod, 1993; Garcia-Fernàndez *et al.*, 1995; and Schuck, 2004, Undergraduate Thesis, Universidade do Vale do Rio dos Sinos, São Leopoldo).

Starting with the casual identification in our laboratory of an MLE in *Girardia schubarti*, we decided to investigate the presence of this element in other planarian species by using such techniques as PCR, Southern Blot, and the hybridization of genomic DNA in the dot blot. Furthermore, the dot blot was also used to estimate the copy number rate per haploid genome of several species.

## Material and Methods

###  Species and DNA extraction

In this study, twenty-eight individuals from 15 planarian species and from several locales in the state of Rio Grande do Sul (southern Brazil) were analyzed, these being the terrestrial planarians *Choeradoplana *sp., *C. iheringi*, *Choeradoplana *sp. 249, *Geoplana *sp. 1759, *G. franciscana*, *G josefi*, *G. ladislavii*, *Notogynaphallia abundans*, *N. ciciliae*, *N. maginata sensu graffi* and *Pasipha* sp., and the freshwater ones *Girardia anderlani*, *G. biapertura*, *G. schubarti* and *G. tigrina* (Table 1). All the freshwater specimens had been collected over the previous 20 years, having been kept in the laboratory at the Institute for Planarian Research (UNISINOS). Prior to DNA extraction, all the individuals were starved during one week. The DNA was extracted by proteinase K, SDS and phenol-chloroform, using standard procedures (Sambrook and Russell, 2001).

###  PCR amplification

PCR was performed on 100 ng of genomic DNA from each tested individual. The degenerate primers had been established beforehand by Robertson (1993), based on the two conserved motifs of the *mariner* transposase, WVPHEL (forward) and YSPDLAP (reverse) (Figure 1). They were termed MAR124F (5' TGGGTNCCNCAYG ARYT 3') and MAR276R (5' GGNGCNARRTCNGGN WSRTA 3'), respectively. A 30 μL-reaction included 2.5 mM MgCl_2_, 0.2 mM dNTP, 0.8 μM of each primer and 1 unit of *Taq* polymerase. After an initial denaturation for 2 min at 95 °C, the PCR condition consisted of 30 cycles of 1 min denaturation at 95 °C, 1 min annealing at 50 °C and 1 min extension at 72 °C, followed by an extra 5 min extension at 72 °C. The predicted size of the PCR products was approximately 500 bp.

In order to identify the two different *mariner* subfamilies *mellifera* and *cecropia*, semi-nested PCRs were performed using three reverse primers separately combined with an MAR124F primer (for primer localization, see Figure 1). The MELLIFERA (5' ATNCCYTTRTARTCCC ACCA 3') primer was used for the *mellifera* subfamily, and both CECROPIA (5' TARTGNAYNACNCCNGC 3') and TIGRINA (5' TARTGDATNACNCCRTA 3') for the *cecropia*. MELLIFERA and CECROPIA primers had already been designed and tested by Robertson (1993) as MAR124F and MAR276R primers, the former being specific for amplifying these two subfamilies. We ourselves designed the TIGRINA primer based on sequence results of Garcia-Fernàndez *et al.* (1995) for *Girardia tigrina*, with the intention of reducing CECROPIA primer degeneracy. 2 μL of the MAR124F/MAR276R PCR amplification were used for each semi-nested PCR, under the same reaction and cycling conditions as described above. The expected size of the semi-nested products was 250 bp approximately.

###  Southern and dot blot

PCR and semi-nested PCR products of all individuals were subjected to gel electrophoresis in 1% agarose and transferred to nylon membranes (Hybond N^+^, GE Healthcare) after denaturation.

For estimation of the number of MLE copies from the *cecropia* subfamily, we chose 20 individuals from the total analyzed by Southern blot (28), and blotted their genomic DNA onto a Hybond N^+^ membrane using a vacuum manifold (Gibco-BRL). Firstly, all DNAs were visually quantified in a UV transilluminator from several different dilutions electrophoresed on 0.8% ethidium bromide stained agarose gels. Following this first estimate, the concentration of all samples was adjusted to 50 ng/μL and further quantified for greater accuracy. DNA samples were then diluted with a TE (10 mM Tris, 1 mM EDTA, pH 8) buffer, in order to obtain the amounts of 800, 400, 200 and 100 ng of genomic DNA in a volume of 20 μL. Each 20 μL of the sample was denatured at 95 °C for 5 min and immediately blotted onto the nylon membrane. For five individuals, fewer dilutions were prepared due to limitations in available DNA.

The membranes were hybridized to a random primer fluorescein labeled probe at 60 °C. The final washing was at 60 °C with 0.5 X SSC, 0.1% SDS. Hybridization and detection were undertaken using a Gene Images Kit (GE Healthcare), according to manufacturer's instructions. A 0.3 kb PCR product containing the central part of a MLE, in which the beginning is from the coding sequence for the WVPHEL conserved motif, was used as a probe. This product was obtained by PCR amplification, using M13 forward and reverse primers from a clone previously produced in our laboratory (Schuck, 2004, Undergraduate Thesis, Universidade do Vale do Rio dos Sinos, São Leopoldo), and containing a MLE *cecropia* subfamily from *Girardia schubarti* inserted into the pGEM T-Easy vector (Promega). After amplification, the product was gel purified using a Wizard SV Gel and PCR Clean-Up System Kit (Promega) before labeling.

1D Image Analysis software version 2.0.3 (Eastman Kodak Company) was used to evaluate the intensity of the hybridization signal and estimate the abundance of MLEs. The estimate was standardized by hybridization signals from 5 ng, 1 ng, 100 pg and 10 pg of the 0.3 kb fragment used as a probe, and by assuming that the complete MLE (one copy) in the genome has 1.3 kb. The assumed size of the *Girardia tigrina* haploid genome was 1.4 x 10^9^ bp, as defined by flow cytometry and reported by J. Baguña (Animal Genome Size Database). We used this value to extrapolate the size of the haploid genome in the other species according to the number of chromosomes each presented.

###  Karyotyping

In order to confirm the haploid number of chromosomes in various species, so as to adjust the estimate of the number of copies of MLE per haploid genome, we karyotyped three individuals of both *Girardia schubarti* and *G. tigrina* species from clonal populations. Ten mitotic cells from each individual were analyzed to determine the ploidy level. Karyotypes were derived from regenerated blastemas according to a modified Hochberg and Erdtmann (1988) protocol. Three days after feeding, the planarians were cut up and then left for three days to regenerate. After colchicine treatment (0.2% for 3 h at room temperature), the tissue was hypotonically shocked in a 75 mM KCl solution containing 2-6 drops of a 0.03% trypsin solution, and then fixed in a glacial acetic acid/methanol solution (1:3). A 10% Giemsa solution was used for chromosome staining. Slides were examined and photographed through a Zeiss Axioskop microscope.

We used data from Benya *et al.* (2007) and Knakievicz *et al.* (2007) for the chromosome numbers of *G. anderlani* and from Benya *et al.* (2007) for *G.biapertura*, since these researchers had karyotyped individuals from the same clonal populations we have worked, as well as for *G. schubarti* and *G. tigrina*.

## Results

###  MLE occurrence

Twenty-eight specimens from 15 species of planarians (four freshwater and 11 terrestrial types) were analyzed by the dot blot and/or PCR and Southern blot techniques to detect the presence of MLEs. The species were collected at six different locales. All the species analyzed, as well as all the collection sites, manifested positive results as to the presence of a MLE in at least one of the techniques employed (Table 2).

After amplification with the MAR124F/MAR276R primers, all the freshwater species analyzed (*Girardia anderlani*, *G. biapertura*, *G. schubarti* and *G. tigrina*), besides two of the terrestrial species (*Choeradoplana iheringi* and *Geoplana ladislavii*), presented visible amplification products of the expected size in agarose gel. The hybridization of these PCR products confirmed the same positive result for all the freshwater species. Although amplification occurred in only two terrestrial species, as stated, hybridization was positive this also being the case of two others - *Geoplana josefi* and *Notogynaphallia maginata sensu graffi*.

Apart from *G.tigrina*, amplification with MAR124F/MELLIFERA primers presented positive results, both for all the species analyzed and for the great majority of specimens. The quantity of products was greater, mainly in the freshwater species, but also for the terrestrial species *Choeradoplana *sp. (CspAS) and *C. iheringi* 2 (Ci2AS). The same result was obtained in the hybridization of PCR products from the terrestrial species. The two individuals that did not present amplification products (Gj2AS and GtGr) also showed no signs of hybridization.

As regards the primer pair MAR124F/TIGRINA which amplifies elements of the *cecropia* subfamily, very weak amplification was observed in only six individuals belonging to the terrestrial species *C. iheringi*, *N. abundans*, *N. ciciliae* and *N. maginata sensu graffi*. Hybridization was, however, positive for fourteen of the nineteen specimens analyzed. Amongst the freshwater species all the specimens were positive for both amplification and hybridization. Only in the terrestrial species *Geoplana* sp. 1759 and *Pasipha* sp. were results unpositive for the presence of *cecropia*, and this when using the TIGRINA primer in the semi-nested PCR.

As a result of amplification with the MAR124F/CECROPIA primers, which, as is the case of TIGRINA, are also used to identify the *cecropia* subfamily, we observed positive results only for specimens of *C. iheringi* among the terrestrial species. Hybridization was positive, however, for twelve of the nineteen analyzed and belonging to seven different species. When using this technique (semi-nested PCR followed by hybridization), only *Geoplana *sp. 1759 and *Pasipha* sp. were negative for occurrence of the *cecropia* subfamily. Results, when using both pairs of primers for identifying this subfamily in the terrestrial species, were very similar, the only difference being the positive hybridization signal observed in *Geoplana franciscana* and *G. ladislavii*, and this when using only the TIGRINA primer. Among the freshwater species, all the specimens presented amplification products visible in gel, except for *G. tigrina* from Gramado (GtGr), although all were positive for hybridization.

As a rule we observed that in the freshwater species, and when using different primer pairs, there was an improvement in amplification and hybridization signals were more pronounced, when compared with the terrestrial species (Table 2). Of all the primer pairs, MAR124F/MELLIFERA produced the best results for amplification, although hybridization was more intense for products of the *cecropia* subfamily, which is explained by our using a *cecropia* probe and highly stringent conditions.

The *Geoplana ladislavii* species was the only one to present wider amplification bands and hybridization signals with MAR124F/MAR276R primers than with those specific for subfamilies. These results were contrary to what was observed with the remaining species, which presented bands and signals that were identical or more intense for at least one of the subfamilies with specific rather than MAR124F/MAR276R primers.

We observed differences among specimens of *G.josefi*, all from the same location. Hybridization occurred with the MAR124F/MAR276R product in only one of the five specimens analyzed (Gj2AS), although neither amplification nor hybridization with the other primers was observed. Two of the three analyzed specimens from São Francisco de Paula (Gj3SF and Gj5SF) presented hybridization with primer products which amplified the *cecropia* subfamily, whereas the third (Gj4SF) presented no positive signal for this subfamily. Nevertheless, the presence of the *mellifera* subfamily was identified in all the three. In the case of the specimen of *Girardia anderlani* from the same locale (São Francisco de Paula), we found amplification and hybridization with the products from all the primer pairs used.

### *cecropia* MLE copy number rate per haploid genome

**Figure 2:**
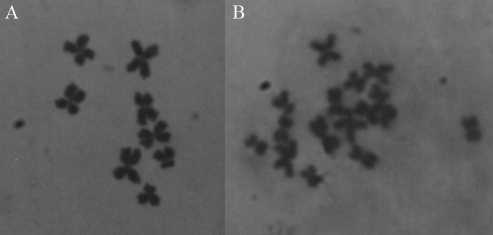
Karyotype of (A) *Girardia schubarti* (2n = 8) and (B) *G. tigrina* (2n = 16).

In order to evaluate ploidy and establish its relationship with estimates of the number of copies of the MLE per haploid genome, the karyotypes of two specimens were analyzed, one from the species *Girardia schubarti* and the other from *G. tigrina.* Thus, it was possible to confirm the number of chromosomes in the lineages analyzed and already described in previous works (Benya *et al.*, 2007; Knakievicz *et al.*, 2007).

Regarding *G. schubarti*, we observed eight chromosomes per cell in all the slides and sixteen in *G tigrina* (Figure 2), from which it was assumed that n = 4 and n = 8 were the respective sizes of the haploid genomes for the other specimens of these two species when calculating the number of copies per haploid genome (for n = 4 and n = 8 the genome size is around 0.7 x 10^9^ and 1.4 x 10^9^ bp, respectively). In the case of *G. anderlani*, we considered n = 9, as previously described by Benya *et al.* (2007) and Knakievicz *et al.* (2007), and the same for *G. biapertura*, as described by Benya *et al.* (2007) (for n = 9, the genome size is around 1.6 x 10^9^ bp). The presence of MLEs in the 15 species analyzed was confirmed by hybridization in all the 20 samples analyzed by dot blot (data not shown). As to the freshwater species, which, besides having a known karyotype, were considered as diploids, the difference in intensity of the hybridization signal was used to estimate a rate for the number of *cecropia* subfamily *mariner* copies. We observed a wide range within the genus *Girardia* - *G. biapertura*, with an extremely high number of copies, about 3 x 10^4^, and, on the other hand, *G. schubarti* from Constantina (Gs5Co) with about 10^3^ copies per haploid genome. Nevertheless, most *Girardia* species presented a rate of 10^4^.

These results were obtained when considering diploid freshwater planarian individuals/populations. However, when we estimated the number of copies considering 3n individuals, the same rate of 10^3^ to 10^4^ copies was maintained.

There are no reports published on terrestrial planarian karyotypes. In an attempt to infer the approximate number of *cecropia* MLE, we assumed n = 8 (haploid genome size = 1.4 x 10^9 ^bp), since it is the most frequently observed chromosome number per haploid genome for both freshwater and terrestrial triclads from Rio Grande do Sul (A.M. Leal-Zanchet, personal communication). So, according to this assumption, we deduced that, as for *Girardia biapertura*, *Choeradoplana* sp. 249 also presented an extremely high number of *cecropia* MLE copies, with about 3 x 10^4^, whereas *Geoplana ladislavii*, *N. abundans* and *N. maginata sensu graffi* from São Francisco de Paula presented a little more than 4 x 10^3^. The other species of the genus *Choeradoplana*, *C. iheringi* and *Choeradoplana* sp*.*, together with *N. ciciliae* and *N. maginata sensu graffi* from Aparados da Serra, and *Geoplana josefi*, presented a rate of 10^4^.

## Discussion

All the planarian species analyzed herein presented positive results for the presence of the *mariner*-like element. This is the first report on MLE in terrestrial planarians, the results implying that MLEs are widely distributed in triclads. In contrast, Garcia-Fernàndez *et al.* (1995) analyzed 10 species of planarians and detected MLEs only in *Girardia tigrina*, this leading them to suggest that this was one of the few species in the group to present the element. We believe that a possible cause for the absence of PCR amplification in the species analyzed by said authors is their use of primers based on the sequence of inverted terminal repeats, which they had found in the MLE from *G. tigrina* and belonging to the *cecropia* subfamily. It is known that these sequences may present wide variability within and among subfamilies (Bigot *et al.*, 2005), and with the PCR technique it is always possible that the primers do not anneal due to certain variation in the sequence of the organism in question. Furthermore, in the case of planarians, the excess of polysaccharides in the mucus may interfere with the efficiency of amplification (Ausubel *et al.*, 1994; Angeles *et al.*, 2005).

Robertson (1997), the author who had previously established the MAR124F/MAR276R primer pair for identifying the *mariner*-like element based on very conservative motifs (Robertson, 1993), obtained positive results from the analysis of *G. tigrina*, *Stylochus zebra* and *Bdelloura candida* planarians. These primers have been widely used for the most diverse organisms (Robertson, 1993, 1997; Robertson and MacLeod, 1993; Augé-Gouillou *et al.*, 1995; Kumaresan and Mathavan, 2004), the results demonstrating that they are both reliable and do not promote false-positive amplifications. Therefore, although we did not sequence the PCR products, the proven reliability of MAR124F/MAR 276R primers and the hybridization of the samples with the *cecropia* probe under highly stringent conditions, allowed us to assume that the amplification products and the hybridization signals did, in fact, represent MLEs in the genome of the individuals analyzed.

Unlike freshwater species, most terrestrial species did not express amplification with MAR124F/MAR 276R primers. As mentioned beforehand, the absence of amplification could be due to the poor quality of the DNA. The samples of terrestrial species, especially from the genera *Geoplana* and *Pasipha,* were considerably viscous. Another possible, more reasonable explanation for the absence of amplification may be the lesser homology of the MAR276R reverse primer with the terrestrial species, seeing that, as a result of amplification with reverse primers specific for the *mellifera* and *cecropia* subfamilies, all the samples presented positive results for one or both. The greatest discrepancy in results was found in Gj2AS, which presented positive hybridization with the products from MAR124F/MAR276R primers, but manifested neither hybridization nor amplification for the two subfamilies analyzed, in spite of the positive dot blot signal. This suggests that the element present in this individual is similar to *cecropia* but with a divergence in the sequence of nucleotides in the annealing region of the specific primers.

On analyzing the results of subfamilies, we observed that only two species, *Geoplana* sp. 1759 and *Pasipha* sp., were positive only for the *mellifera* subfamily. This result was also found for two of the *Geoplana josefi* specimens analyzed (Gj1AS and Gj4SF), in spite of the presence of the *cecropia* subfamily having been identified in the other two specimens of this same species from São Francisco de Paula (Gj3SF and Gj5SF). Furthermore, only *cecropia* was found in *Girardia tigrina* from Gramado (GtGr). In all the other species, results were positive for the presence of the two subfamilies under investigation (conclusions for *mellifera* were arrived at based only on specific semi-nested PCR and weak hybridization with a *cecropia* probe). These results indicate the presence of these subfamilies in the positive cases, but do not exclude the possibility that a larger number of MLEs exists, including those belonging to other subfamilies. The identity of sequences in different subfamilies is 40%-56% at the DNA level and 23%-45% in amino acids (Robertson, 1993). This probably explains the low intensity of hybridization of the probe with amplification products of the *mellifera* subfamily, notwithstanding the large quantity of product visible in gel. On the other hand, products for the *cecropia* subfamily in small quantities or not even at all visible (in most of the terrestrial species), gave signs of intense hybridization. Thus, it is possible that other MLE subfamilies could be present in these planarians, since we probed only those genomes with *cecropia* and used stringent hybridization conditions.

Elements of the *mariner* family have considerable capability for occupying one and the same genome with more than one subfamily. In some cases up to nine distinct types of *mariner* have been observed in a single genome (Lampe *et al.*, 2001). The subfamilies most frequently found are *cecropia*, *mauritiana*, *mellifera*, *irritans* and *capitata* (Robertson and MacLeod, 1993). Robertson (1997) identified clones belonging to several subfamilies in *G. tigrina* - *cecropia*, *mellifera*, *mauritiana*, *capitata* and *lineate -*. This confirmed what we ourselves found for this species on considering the two subfamilies we analyzed.

An estimate of the number of copies of *cecropia* MLEs varied from 10^3^ to 10^4^ per haploid genome of the species analyzed. Considering the high stringency conditions for hybridization when using a *cecropia* probe, it is possible that these values are underestimated for the *marine*r family, since elements other than those of the *cecropia* subfamily could also be present. On the other hand, assuming that all analyzed individuals were diploids, although they could be polyploids, we may be overestimating the number of *cecropia* copies per haploid genome, seeing that these individuals may present some of the chromosomal variations usually described for this group. However, the change from diploid to triploid for example, the most common situation encountered, does not alter the 10^3^ to 10^4^ variation rate.

It is important to point out that, even though the dot blot is still being used to determine the copy number of transposable elements (Zakharkin *et al.*, 2004; Zhao *et al.*, 2007; Ma *et al.*, 2008; Sun *et al.* 2008), we recognize this to be a limited technique, and thus there is the possibility of under- or over-estimating. Nevertheless, even taking this limitation into consideration, the intensity of hybridization signals was high enough to assert that we have found a higher number of *mariner* copies in a planarian group than has ever been reported before.

Eight thousand copies per haploid genome have been reported for the species *G. tigrina*, which is considered to be a high number, covering approximately 0.7% of its genome (Garcia-Fernàndez *et al.*, 1995). In our results, we observed the presence of 13,000 to 18,000 copies in the same species, a higher number than any previously reported, and which represents approximately twice the percentage of the described genome. This value, considered high by Garcia-Fernàndez *et al.* (1995), ended up by being contrasted with the much higher value we found for *Choeradoplana* sp. 249 and *Girardia biapertura*, in which around 3 x 10^4^ copies were estimated for their genomes. This number is only exceeded by the 44,000 *mariner* copies estimated for the genome of the insect *Forficula auricularia* (Barry *et al.*, 2004).

From our results, we concluded that, with the exception of *Geoplana sp*. 1759 and *Pasipha sp.,* at least two subfamilies were present in the planarian genomes analyzed, and that the majority of the individuals analyzed presented a large number of copies. Jacobson *et al.* (1986) suggest that the transposition mechanism in species that present large numbers of copies, such as *G. tigrina,* is similar to the *Mos* factor of *Drosophila mauritiana*, which is capable of inducing not only its own transposition, but also that of other *mariner* elements belonging to other subfamilies. In human beings, 1,000 copies of the MLE have been estimated, in mice 20-50, in sheep 3,000 and in cattle 3,500 (Demattei *et al.*, 2000). In the insect *Forficula auricularia*, MLEs represent 4% of its genome, with an estimated 40,000 copies. In another insect, *Ochlerotatus atropalpus*, between 370 and 1,200 copies of *Atmar-1* and from 100 to 300 of *Atmar-2* have been estimated (Zakharkin *et al.*, 2004). These results, demonstrate, as ours do, that the number of copies varies drastically between organisms and populations.

The different number of copies and their distribution may be interpreted as a manifestation of different stages in the evolutionary history of the *mariner* element (Torti *et al.*, 2000). These stages are reflected in the proliferation of the element, whereat an increase in the amount of copies occurs. This increase could cause a reduction in the transposition rate and vertical inactivation, followed by stochastic loss, thereby resulting in a reduction in the number of copies (Hartl *et al.*, 1997b). Lohe *et al.* (1995), when performing the Southern blot with the DNA of various groups of Drosophilae, reported that the signal observed in the hybridization of *Drosophila ananassae* was stronger than expected, since the probe was a fragment of a species belonging to another group of the same genus, *D. erecta*, which is at a certain evolutionary distance. From these results, they suggested that this would be a strong indicator of sequence similarity, thereby representing a case of horizontal transmission. Our finding of a strong hybridization signal in the dot blot for some terrestrial species such as those of the genera *Choeradoplana* and *Notogynaphallia*, which belong to an infra-order different from the one we used the probe on, is a stimulus for us to continue looking for evidence that can give support to the idea of horizontal transmission for the *mariner* element, as is suggested by several authors (Robertson and MacLeod, 1993; Lohe *et al.*, 1995; Hartl *et al.*, 1997a; Robertson, 1997), although it is not known how this transmission occurs.

From the present study, we concluded that the occurrence of MLEs is widespread in the triclad group, as was observed in all the species analyzed, and a very high number of copies, ranging from 10^3^ to 10^4^, were estimated for a vast majority of the species analyzed. These results exceeded our expectations, both as to occurrence as well as numbers, and suggest that the MLE is widely distributed in the planarian group.

## Figures and Tables

**Table 1:** Abbreviations used for the individuals investigated in the present study and their geographical origin.

	Individual^a^	Species	Origin^b^
	GaSF	*Girardia anderlani*	SF
	GbSS	*Girardia biapertura*	SS
	Gs1SF	*Girardia schubarti* individual 1	SF
	Gs2Gr	*Girardia schubarti* individual 2	Gr
Fresh water	Gs3Co	*Girardia schubarti* individual 3	Co
	Gs4Co	*Girardia schubarti* individual 4	Co
	Gs5Co	*Girardia schubarti* individual 5	Co
	Gs6Co	*Girardia schubarti* individual 6	Co
	GtGr	*Girardia tigrina*	Gr

	C249SF	*Choeradoplana* sp. 249	SF
	Ci1SF	*Choeradoplana iheringi* individual 1	SF
	Ci2AS	*Choeradoplana iheringi* individual 2	AS
	CspAS	*Choeradoplana* sp.	AS
	G1759AS	*Geoplana* sp. 1759	AS
	GfSF	*Geoplana franciscana*	SF
	Gj1AS	*Geoplana josefi* individual 1	AS
	Gj2AS	*Geoplana josefi* individual 2	AS
Terrestrial	Gj3SF	*Geoplana josefi* individual 3	SF
	Gj4SF	*Geoplana josefi* individual 4	SF
	Gj5SF	*Geoplana josefi* individual 5	SF
	GlSF	*Geoplana ladislavii*	SF
	NaIp	*Notogynaphallia abundans*	Ip
	Nc1SF	*Notogynaphallia ciciliae* individual 1	SF
	Nc2SF	*Notogynaphallia ciciliae* individual 2	SF
	Nm1SF	*Notogynaphallia maginata sensu graffi* individual 1	SF
	Nm2SF	*Notogynaphallia maginata sensu graffi* individual 2	SF
	Nm3AS	*Notogynaphallia maginata sensu graffi* individual 3	AS
	PspAS	*Pasipha* sp.	AS

^a^Letters and numbers used for abbreviations refer to taxonomic names, numerical values when there are more than one individual of the same species, and geographical origin. ^b^Geographical origin: SF - National Forest of São Francisco de Paula (29° 26' 53” S, 50° 35' 01” W); SS - São Sebastião do Caí (29° 35' 12” S, 51° 22' 32” W); Gr - Gramado (29° 22' 43” S, 50° 52' 26” W); Co - Constantina (27° 44' 05” S, 52° 59' 32” W); AS - Aparados da Serra National Park (29° 02' 52” S, 50° 08' 41” W); Ip - State Park of Itapuã (30° 16' 55” S, 51° 01' 12” W).

**Table 2:** PCR amplification and intensity of hybridization (H) with a *cecropia* probe in Southern and dot blot. Primers used: MAR124F/MAR276R; MELLIFERA, TIGRINA and CECROPIA were reverse primers used in a semi-nested PCR combined with MAR124F.

	Individual	MAR124F/ MAR276R			MELLIFERA			TIGRINA			CECROPIA		Dot blot
		PCR	H		PCR	H		PCR	H		PCR	H	
	GaSF	++	++		++	++		++	++		++	+++	++
	Gb1SS	++	++		++	++		++	+++		++	+++	++++
	Gs1SF	++	++		++	++		++	++		++	+++	+++
Fresh water	Gs2Gr	++	++		++	++		++	++		++	++	+++
	Gs3Co	++	++		++	++		++	++		++	++	+++
	Gs4Co	++	++		++	++		++	++		++	++	NA
	Gs5Co ^a^	++	+++		++	++		++	++		++	+++	++
	Gs6Co	++	+++		++	++		++	++		++	++	NA
	GtGr ^a^	++	++		-	-		++	++		-	++	+++

	C249SF	-	-		++	++		-	++		-	++	++++
	Ci1SF	-	-		++	++		+/-	++		++	++	+++
	Ci2AS	++	++		++	++		+/-	++		+/-	+++	NA
	CspAS	-	-		++	++		-	++		-	++	+++
	G1759AS	-	-		++	++		-	-		-	-	++
	GfSF	-	-		++	++		-	++		-	-	++
	Gj1AS	-	-		++	++		-	-		-	-	NA
	Gj2AS	-	++		-	-		-	-		-	-	++
	Gj3SF	-	-		++	++		-	++		-	++	NA
Terrestrial	Gj4SF	-	-		++	++		-	-		-	-	NA
	Gj5SF	-	-		++	++		-	++		-	++	NA
	Gl1SF	++	+++		++	++		-	++		-	-	++
	Na1Ip	-	-		++	++		+/-	++		-	++	++
	Nc1SF	-	-		++	++		+/-	++		-	++	+++
	Nc2SF	-	-		++	++		-	++		-	++	+++
	Nm1SF	-	-		++	++		+/-	++		-	++	NA
	Nm2SF	-	-		++	++		+/-	++		-	++	++
	Nm3AS	-	++		++	++		-	++		-	++	+++
	Psp1AS	-	-		++	++		-	-		-	-	++

Positive (+) and negative (-) signals indicate the presence or absence of PCR amplification or hybridization, respectively: (++++) very high hybridization; (+++) high hybridization; (++) large amount of PCR product or intermediate hybridization; (+) small amount of PCR product or weak hybridization; (+/-) very small amount of PCR product; (NA) not analyzed. ^a^individuals used in karyotyping.
